# Purification and identification of a surfactin biosurfactant and engine oil degradation by *Bacillus velezensis* KLP2016

**DOI:** 10.1186/s12934-021-01519-0

**Published:** 2021-01-28

**Authors:** Khem Raj Meena, Rajni Dhiman, Kailash Singh, Sachin Kumar, Abhishek Sharma, Shamsher S. Kanwar, Rittick Mondal, Sandip Das, Octavio L. Franco, Amit Kumar Mandal

**Affiliations:** 1 Department of Microbiology, CBS&H, Dr. Rajendra Prasad Central Agricultural University, Pusa, Samastipur, 848125 Bihar India; 2 Forest Research Institute, Dehradun, 248006 India; 3 Department of Chemistry, Himachal Pradesh University, Shimla, 171 005 India; 4 Department of Zoology, University of Rajasthan, Jaipur, 302 004 India; 5 Department of Biotechnology, Himachal Pradesh University, Shimla, 171 005 India; 6 Chemical Biology Laboratory, Department of Sericulture, North Dinajpur, Raiganj, 733134 West Bengal India; 7 School of Sciences, Netaji Open University, Durgapur, West Bengal India; 8 Post-Graduate Program in Biotechnology, Catholic University Dom Bosco, Campo Grande, Mato Grosso do Sul Brazil; 9 Centro de Análises Proteômicas E Bioquímicas, Programa de Pós-Graduação em Ciências Genômicas e Biotecnologia, Universidade Católica de Brasília, Brasília, Distrito Federal Brazil; 10 Centre for Nanotechnology Sciences, Raiganj University, North Dinajpur, Raiganj, 733134 West Bengal India

**Keywords:** *Bacillus velezensis*, Biosurfactant, CO_2_ estimation, Engine oil, Surfactin

## Abstract

Engine oil used in automobiles is a threat to soil and water due to the recalcitrant properties of its hydrocarbons. It pollutes surrounding environment which affects both flora and fauna. Microbes can degrade hydrocarbons containing engine oil and utilize it as a substrate for their growth. Our results demonstrated that cell-free broth of *Bacillus velezensis* KLP2016 (Gram + ve, endospore forming; Accession number KY214239) recorded an emulsification index (E_24_%) from 52.3% to 65.7% against different organic solvents, such as benzene, pentane, cyclohexane, xylene, *n*-hexane, toluene and engine oil. The surface tension of the cell-free broth of *B. velezensis* grown in Luria–Bertani broth at 35 °C decreased from 55 to 40 mN m^−1^at critical micelle concentration 17.2 µg/mL. The active biosurfactant molecule of cell-free broth of *Bacillus velezensis* KLP2016 was purified by Dietheylaminoethyl-cellulose and size exclusion chromatography, followed by HPLC (RT = 1.130), UV–vis spectrophotometry (210 nm) and thin layer chromatography (R_f_ = 0.90). The molecular weight of purified biosurfactant was found to be ~ 1.0 kDa, based on Electron Spray Ionization-MS. A concentration of 1980 × 10^–2^ parts per million of CO_2_ was trapped in a KOH solution after 15 days of incubation in Luria–Bertani broth containing 1% engine oil. Our results suggest that bacterium *Bacillus velezensis* KLP2016 may promise a new dimension to solving the engine oil pollution problem in near future.

## Introduction

Environmental pollution is currently one of the most serious global issues. Engine oil used in automobiles is hazardous and toxic to the soil. Used engine oil that is spilled or wrongly discarded may enter storm water runoff and eventually enter into water bodies affecting adversely the environmental health of receiving water bodies [1]. Oil spills into the sea is an emerging issue, harming marine flora and fauna [2]. To protect the flora and fauna of the water bodies, treatment of engine oil (main polluting agent) is usually required.

Various treatment procedure involving both chemical and physical methods, like dissolving, precipitation or absorption, using a range and combination of processes to remove non-hydrocarbons, impurities and other constituents that may severely affect the performance properties of finished products or reduce the efficiency of the conversion processes. Methods comprise separation or removal of aromatics and naphthenes, including impurities and undesirable contaminants. Before processing Sweetening compounds and acids are used to desulphurize crude oil. Other treatment methods include chemical sweetening, crude desalting, clay contacting, acid treating, solvent refining, hydrodesulphurizing, caustic washing, drying, hydrotreating, solvent dewaxing and solvent extraction [3, 4].

Different types of chemicals are used in petroleum industries for various operations, mainly in oil recovery [5]. The process releases contaminants and causes water contamination, posing health risks to living beings [6, 7]. Recalcitrant hydrocarbon (present in engine oil) degrading microbes of genus *Bacillus* produce biosurfactants of a diverse chemical nature and molecular size, with different active role(s). These microbial biosurfactants have the capability to degrade hydrocarbons enhancing the bioavailability of hydrophobic organic compounds in engine oil [8]. In recent era, biosurfactants have received special attention due to their unique properties like biodegradability, eco-friendly and low toxicity [9]. Biosurfactants sometimes also referred to as ‘green products’ or ‘greener compounds’ for playing a pivotal role in agriculture and cleaning up the environment [8]. The function of the biosurfactants is to emulsify the non-aqueous phase liquid contaminants and to increase its solubility. These features of biosurfactants facilitate contaminants export from the solid phase and allow the microorganisms adsorbed on the soil particles to access and remove the contaminant molecule [10–12]. They also have the capacity to generate a renewable source of energy from cheaper substrates [13]. Biosurfactants produced by microbes have been studied extensively for their role in engine oil degradation and in reducing the risk from various environmental pollutants [5, 14]. The structure–function regarding properties of the microbial biosurfactants attracts various research to explore their potential in bioremediation [15]. Surfactin and iturin are already known to be efficient biosurfactants for degrading hydrocarbons containing engine oil [16]. The important properties making the biosurfactants special are biodegradability, lower toxicity, bioavailability, high foaming, high selectivity and specific activity at extreme temperature, pH and salinity [17, 18].

In this investigation, the main purpose is to report a cost-effective solution towards engine oil degradation by a biosurfactant produced by *B. velezensis* KLP2016 (Gram +ve, endospore forming; Accession number KY214239). The biosurfactant was further purified and characterized, and the engine oil degradation was investigated by GC–MS [19]. This study will be useful for cleaner understanding of both environmental and industrial problem in near future [20].

## Materials and methods

### Production of biosurfactant by *B. velezensis* KLP2016 cells

A fresh loopful culture of *B. Velezensis* KLP2016 was inoculated in 100 mL of Luria-Bertani broth and incubated at 200 rpm under shaking at 30 ºC to get 1.0 OD of cells at 620 nm. For production of biosurfactants, 1000 mL of LB broth was prepared in which 4% (v/v) of bacterial inoculum (1.0 O.D cells) was inoculated and the flasks were incubated for 72 h at 30 ºC at 200 rpm. After incubation, the culture broth was centrifuged at 10,000 rpm for 10 min at 4 ºC [20]. Biosurfactants containing supernatant/ cell free broth was collected for further experiments.

### Measurement of emulsification index, surface tension and critical micelle concentration

Biosurfactant containing culture broth was evaluated by measuring the emulsification index (E_24_%) using various organic hydrocarbon compounds (benzene, pentane, cyclohexane, xylene, *n*-hexane, toluene and engine oil) as the substrate. In a test tube, 1.5 mL of each hydrocarbon compounds was added individually to 1.5 mL *B. velezensis* cell-free broth. The mixture was mixed by using a vortex for 2 min, and the content was left undisturbed for 24 h. The percentage of the emulsification index (E_24_%) was calculated by using the following equation [14].

$${\text{E}}_{24} \left( \% \right) = \frac{{{\text{ Total height of the emulsified layer }}\left( {{\text{mm}}} \right){ }}}{{{\text{Total height of the liquid layer }}\left( {{\text{mm}}} \right)}} \times 100$$

The surface tension of cell-free broth of *B. velezensis* bacterium was calculated in both Luria–Bertani broth and Minimal Salt medium (MSM) individually at 25 °C and 35 °C via using drop weight method [21]. The uninoculated LB and MSM broth (g/l) (KH_2_PO_4_, 1.4; Na_2_HPO_4_, 2.2; (NH_4_)_2_SO_4_, 3; MgSO_4_, 0.6; NaCl, 0.05; yeast extract, 1; CaCl_2_ 0.02) was taken as negative control. Critical micelle concentration (cmc) is the concentration of biosurfactant above which micelle form and further no reduction in surface tension occurs was also determined. The surface tension (γ) and critical micelle concentration (cmc) was calculated by using the following equation [21];

$$\gamma = \frac{{\gamma^{0} n^{0} \rho }}{{np^{0} }}$$

where *γ*^*0*^ is surface tension, *n*^*0*^ is number of drops and *ρ*^*0*^ is the density of uninoculated broths, while *γ* is surface tension, *n* is number of drops and *ρ* is density of cell-free fermentation broth.

### Purification and identification of active compound extracted from the culture broth of* B. velezensis* KLP2016

#### Ammonium sulfate (NH_4_)_2_SO_4_ mediated protein precipitation and dialysis

The cell-free broth was introduced with 0–20, 20–40, 40–60, 60–80 and 80–100% saturation of (NH_4_)_2_SO_4_ at 4 °C, further mixed and kept overnight at 4 °C. Thereafter, the precipitates were deposited after centrifugation at 12,000 rpm for 15 min. The precipitates were reconstituted in 1 mL of 20 mM sodium phosphate buffer at pH 7.5 and checked for emulsification activity against engine oil. One unit of emulsifying activity was explicated as the quantity of emulsifier that yielded an absorbance (600 nm) of 0.1 in the assay mixture [22].

### Ion exchange chromatography

The DEAE cellulose packed glass column (height 10 cm; diameter 1.5 cm) was equilibrated with 20 mM sodium phosphate buffer (pH 7.5) after activation by 0.5 M NaOH. Five mL of dialyzed biosurfactant preparation (4.0 mg protein) was loaded on the matrix in the column [23]. Column was equilibrated with 20 mM sodium phosphate buffer (pH 7.5). Unbound proteins were eluted with low ionic strength buffer (sodium phosphate buffer; pH 7.5) at a flow rate of 1 mL/min and discarded. The bound biosurfactant molecules eluted with the stepwise gradient of 0.5 M NaCl, 1 M NaCl and 1.5 M NaCl in sodium phosphate buffer (pH 7.5; 20 mM), respectively [24]. Emulsification activity and A_280_ values were evaluated against the engine oil.

### Size exclusion chromatography

Sephadex G-25 packed matrix was washed off with several column volume of 20 mM sodium phosphate buffer (pH 7.5).Pooled active fraction of the DEAE was loaded on the bed surface of Sephadex G-25 column and eluted with the sodium phosphate buffer (20 mM; pH 7.5) and fractions were collected [23]. Absorbance at 280 nm and emulsification activity was evaluated against the engine oil. Active fractions were further checked with UV–vis spectrophotometer and TLC, as mentioned below.

### TLC and UV–VIS spectrophotometry

The fractions obtained from size exclusion chromatography, were analysed and mixed on the basis of their OD. A solvent system of chloroform: methanol: water (39:15:3; v/v) was prepared, and 5 µl sample of mixed biosurfactant fractions was applied at the point of origin of the TLC plate [25]. Lipid moiety of the molecule was detected by TLC plate sprayed with water and thereafter kept for drying. The R_f_ values of the biosurfactant spot on the TLC plate were evaluated using the following formula and results recorded accordingly.

$$Rf = \frac{{Distance\,travelled\, by\, the\, solute\, \left( {cm} \right)}}{{Distance\, travelled\, by \,the\, solvent \,\left( {cm} \right)}}$$

The purified biosurfactant was also analysed for ultraviolet spectral analysis [26] at range of 190–800 nm (UV–VIS Spectrophotometer, CARY, VARIAN).

### High performance liquid chromatography analyses

The presence of biosurfactant in the purified molecule was confirmed by HPLC using an HPLC pump (Waters, USA) using a reverse phase column (Lichrosorb C18-5 µm; Merck, Germany) and 2998 photodiode assay detector [20]. The mobile phase contained acetonitrile (ACN): ammonium acetate (10 mM) in the ratio of 40: 60 (v/v) with 2 mL/min flow rate. Biosurfactant sample 5 µl was injected each time and analysed at 254 nm wavelength and compared with standard biosurfactants, *i.e*., surfactin and iturin.

### ESI–MS of purified biosurfactant

A mass spectrometer (Q-TOF micro Waters 2795 UK) was used to find the molecular weight of the purified biosurfactant. The conditions used for MS were temperature source, 100 ºC; 3000 V in positive mode; capillary voltage, cone voltage, 30 V; current source, 80.0 A and capillary voltage of 7.0 V in positive mode [23]. About 20 μl of purified biosurfactant was injected into the MS and gently ionized with CH_3_OH and H_2_O (80:20) using electrospray (ESI) with flow rate of 1.0 mL/min. ESI–MS results were compared with the authentic surfactin biosurfactant molecule to identify the molecular mass of the purified biosurfactant of *B. velezensis*.

### Hydrocarbon degradation activity of *B. velezensis* KLP2016

#### Biodegradation of engine oil (K 15 W-40) by *B. velezensis* KLP2016 in a biometric system

For the biodegradation of engine oil, 5% (v/v) starter inoculum of 7 h of *B. velezensis* KLP2016 culture was inoculated in the 250 mL capacity sterilized flasks each containing 100 mL MSM and LB broth. Hydrocarbon substrate (K 15 W-40 Engine oil) was added at 1% (v/v) concentration in each of the sterilized flasks. The test tubes containing fresh KOH (10 mL; 0.05 M) was placed in each of the flasks, and were incubated at 30 °C under shaking (100 rpm) for 5 to 20 days. The absorbance (A_600_) and CO_2_ content in inoculated and uninoculated broths were monitored at 5-day intervals up to 20 days. The CO_2_ gas trapped in the KOH solution was titrated by introducing 100 µl of barium chloride (w/v; saturated) and three drops of phenolphthalein with 0.05 M HCl until the appearance of the end point as the colourless solution. The difference in millilitres of HCl used to titrate KOH containing solution of control (placebo) and *B. velezensis* KLP2016 inoculated media was converted into ppm of fixed carbon dioxide as described previously [19, 27]. Hydrocarbon degradation of engine oil facilitated by *B. velezensis* KLP2016 cells was also confirmed by Gas chromatography-mass spectrometry (GC–MS).

### Hydrocarbon analysis by GC–MS of K 15 W-40 engine oil treated with *B. velezensis* KLP2016

In order to analyse the hydrocarbon products of engine oil degraded by *B. velezensis* KLP2016, the culture broth (5, 10, 15 and 20 days) was centrifuged at 10,000 rpm, at 4 °C for 10 min. From the supernatant, the upper layer was collected, filtered with syringe filter (0.22 µm) and the filtrate was analysed using GC–MS to evaluate the degraded products. The sample (5 µL) is introduced at flow rate of 1 mL/min. GC–MS analyses was performed using an MS5973 spectrometer with a ULBON HR-1 column (25 mm × 50 mm), with thickness of 0.25 micron, with the carrier gas helium, ion source temperature 230 ºC at 18.5 psi pressure and 20% split ratio [27]. Results were observed and recorded accordingly.

### Statistical analysis

All methods are statistically analysed.

## Results

### Emulsification index, surface tension and critical micelle concentration of biosurfactant containing cell-free broth of *B. velezensis*

**Fig. 1:**
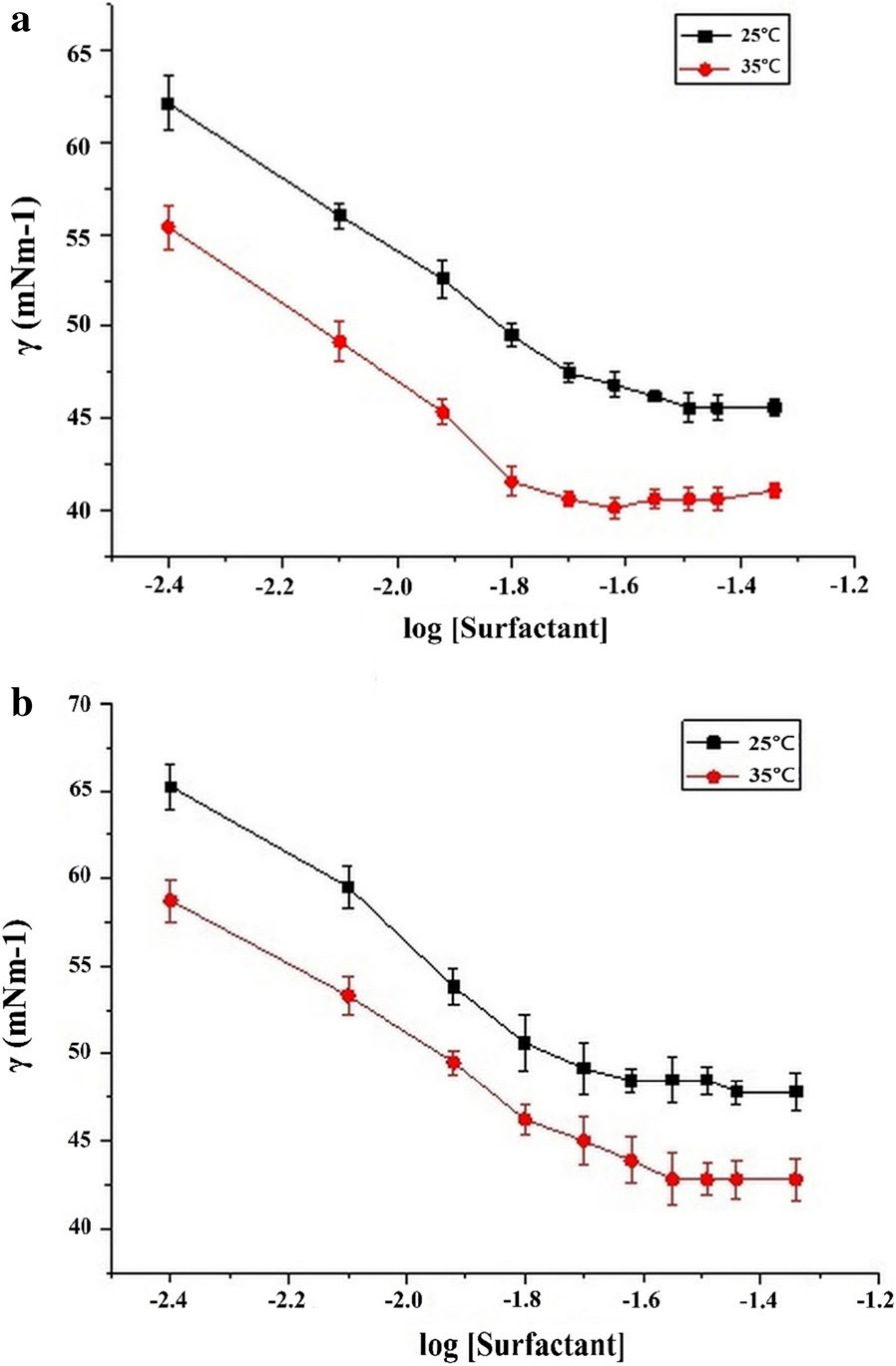
Surface tension and cmc measured against the logarithm concentration of biosurfactant containingcell-free broth. **a** surface tension and cmc of cell-free broth from *B. velezensis* inoculated in LB broth; (**b**) surface tension and cmc of cell-free broth from *B. velezensis* inoculated inMSM broth. Surface tension at 35 ^℃^ was reduced from 55 to 40 mN m^−1^ by cell-free broth of *B. velezensis* grown in Luria Bertani broth

An emulsification index of ≥ 30% was considered as significant emulsification activity. The reported results showed that *B. velezensis* cell-free broth showed E_24_% marked 65.7%, 59.0%, 56.1%, 61.0%, 52.3%, 65.2% and 56.2% with benzene, pentane, cyclohexane, xylene, *n*-hexane, toluene and engine oil, respectively. The surface tension of cell-free broth containing biosurfactant at 35 ºC was reduced from 55 mN m^−1^ to 40 mN m^−1^ at 17.2 µg/mL (cmc) and surface tension at 25 °C was reduced from 62 mN m^−1^ to 48 mN m^−1^ at 17.4 µg/mL of critical micelle concentration in LB broth (Fig. 1a). Whereas, the surface tension at 35 ºC was reduced from 58 mN m^−1^ to 43 mN.m^−1^ at17.6 µg/mL (cmc) and at 25 ºC, surface tension was reduced from 65 mN  m^−1^to 50 mN.m^−1^ at 18.1 µg/mL (cmc) of *B. velezensis* cell-free broth grown in MSM medium (Fig. 1b).

### Purification of biosurfactant by DEAE- cellulose and size exclusion chromatography

**Fig. 2:**
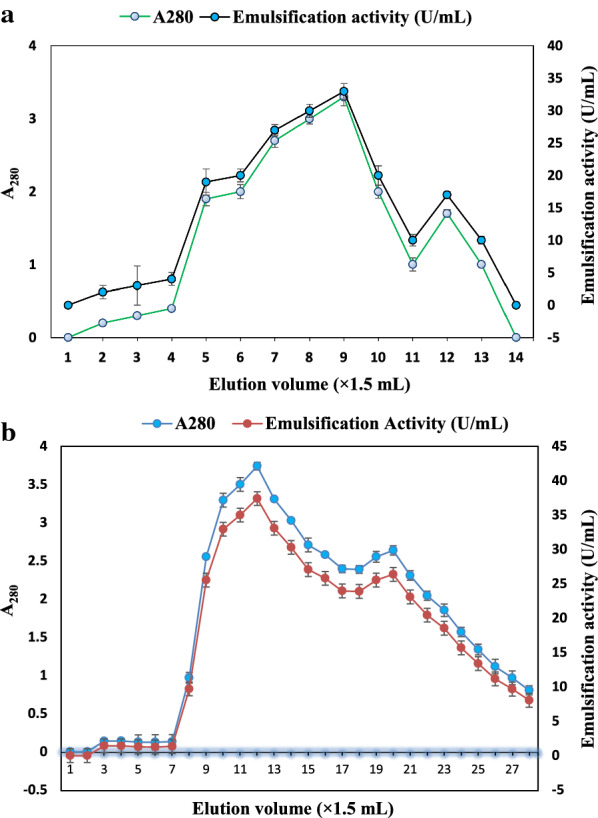
Purification of a biosurfactant and its emulsification activity against engine oil. **a** DEAE columnfractions emulsification activity against engine oil and A_280_; (**b**) Sephadex G-25 column eluted fractions and emulsification against engine oil

**Fig. 3:**
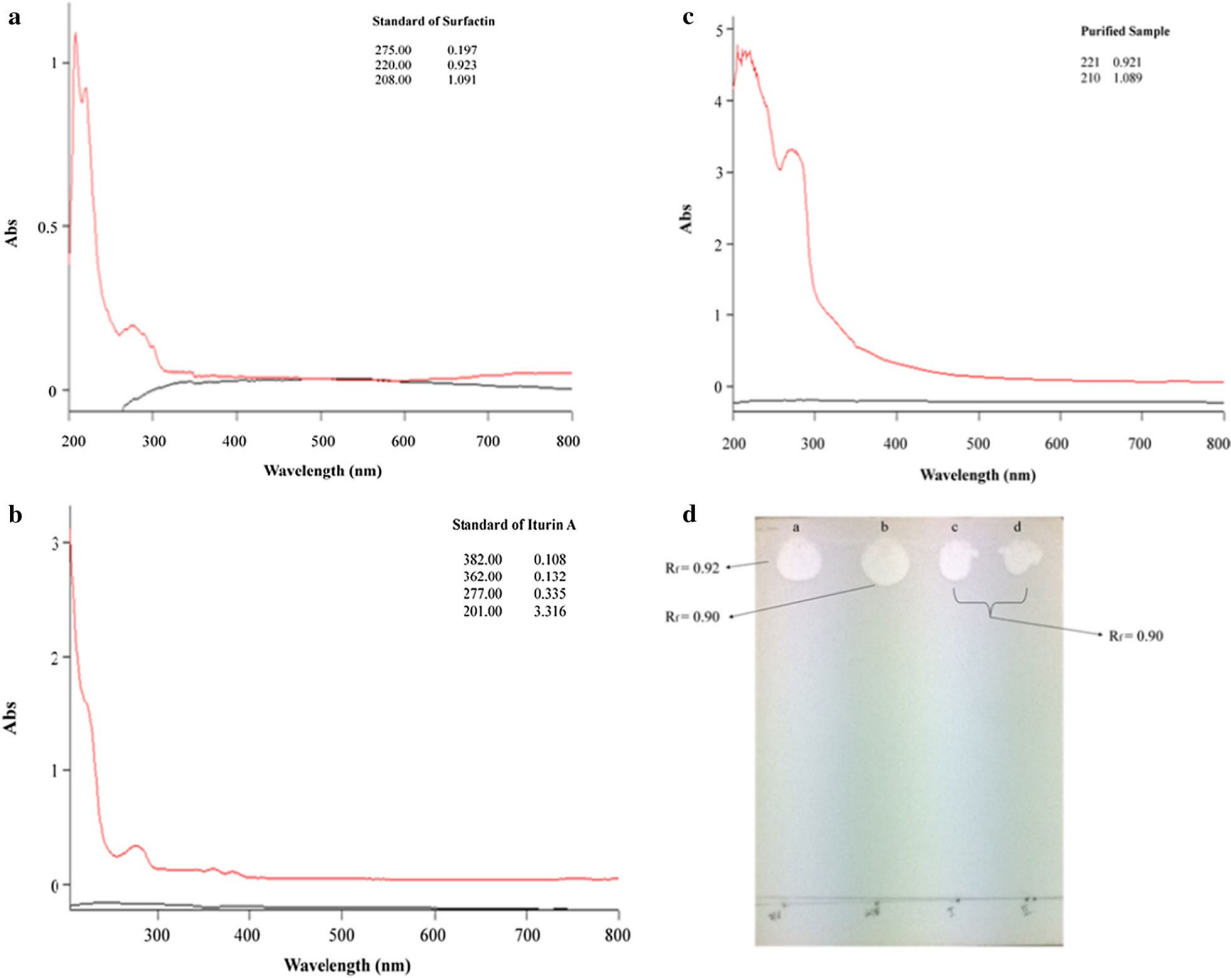
**a** Standard of Iturin A; **b** standard of surfactin; **c** UV visible spectra showing absorbance maxima of biosurfactant containing purified fractions of *B. velezensis* KLP2016at 221 and 210 nm; **d** Spot(s) of the cell-free broth sample and standard onTLC plate, (**a**) iturin, (**b**) surfactin, (**c**, **d**) Purified biosurfactant from *Bacillusvelezensis*KLP2016

On the basis of emulsification activity against the engine oil, an ammonium sulphate cut in the range 20–40% showed 24.0 ± 1.54 U/mL emulsification activity or ~ 60% E24%, was selected for further purification. A total of 15 fractions were collected (1.5 mL each) by elution with 0.5 M, 1 M and 1.5 M NaCl (Fig. 2a). Fractions that were eluted were checked for emulsification activity against engine oil, and the maximum activity was recorded in the case of fraction number 9 (33 U/mL). The active fractions from the DEAE column were collected and loaded on Sephadex G-25 column for further purification. A total of 28 fractions were collected (1.5 mL each) after elution with sodium phosphate buffer. Emulsification activity against engine oil was observed in 9–17 fractions. The fractions (9–17) were checked separately then pooled for further investigations (Fig. 2b). The selected fractions of *B. velezensis* KLP2016 yielded absorbance maxima at 221 and 210 nm, which corresponded to the characteristic absorption of peptide bonds of surfactin (Fig. 3a–c). These results showed that the biosurfactant produced by *B. velezensis* KLP2016 might belong to the ‘iturin or surfactin family’.

### Identification of purified biosurfactant by TLC, HPLC and ESI–MS

**Fig. 4:**
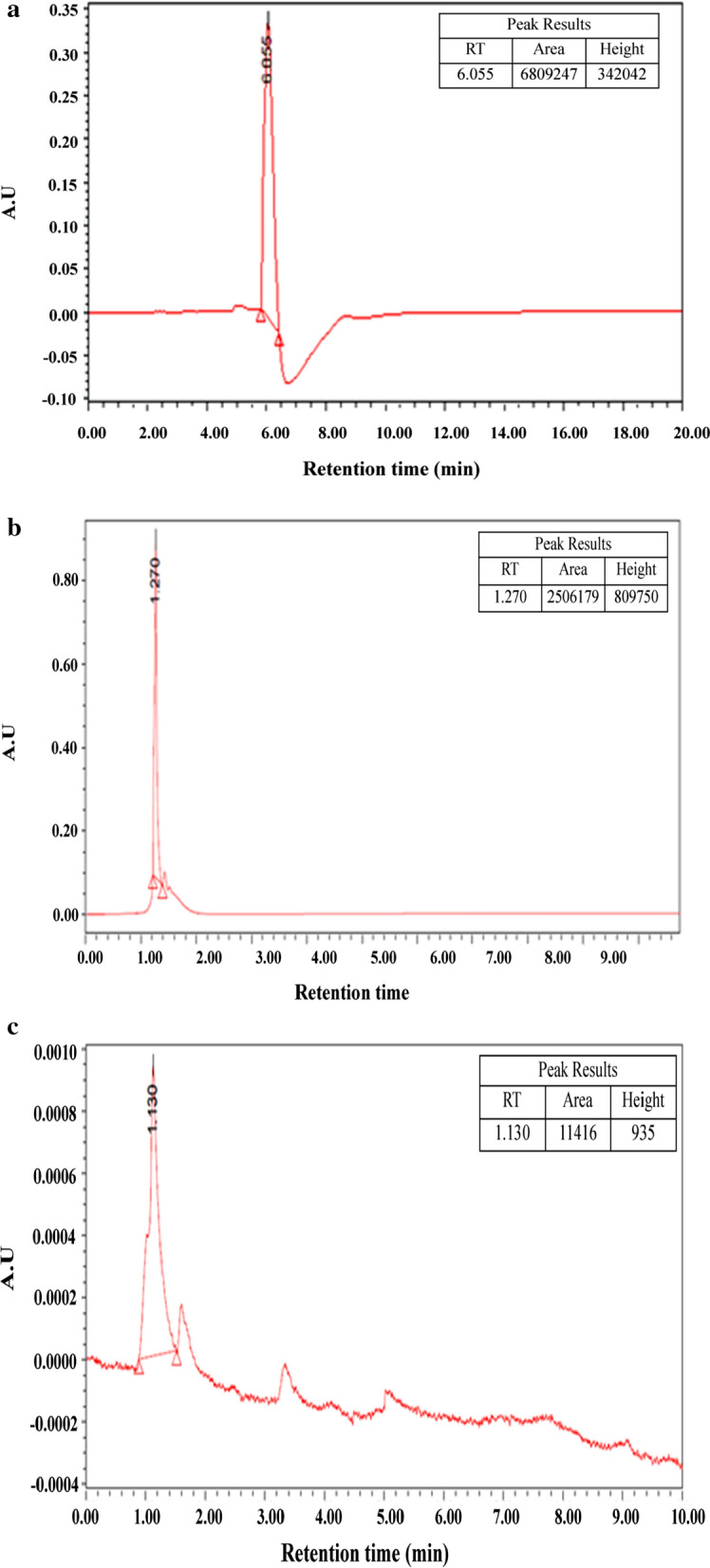
HPLC chromatogram of purified biosurfactant and biosurfactant standards. **a** standard of iturin A; **b** standard of surfactin; **c** chromatogram of active Sephadex G-25 fraction. The purified biosurfactant might be a surfactin showing ~ similar retention time tothat of surfactin

**Fig. 5:**
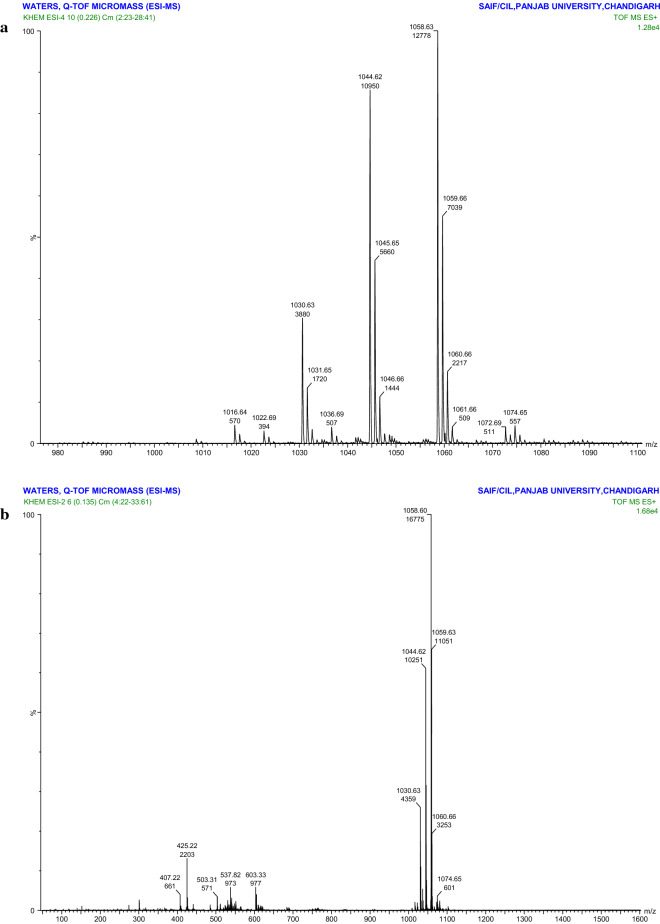
ESI–MS spectra. **a** Standardsurfactin (Sigma Aldrich, USA); and (**b**) purified biosurfactant of *B. velezensis* KLP2016. The peaks of both molecules showed a pattern with adduct of *Mr* (14) of CH_2_ group that indicated the presence of homologues with different carbon length(s)

A white spot was observed when the TLC plate was sprayed with water, indicating the lipophilic nature of the compound (Fig. 3d). Thus, a peptide without free amino groups (cyclic structure) might be present, as assumed after TLC. The standard preparation of surfactin also showed a value 0.94 R_f_, which was similar to the value of 0.90 R_f_ recorded for the biosurfactant, indicating the presence of a surfactin-like biosurfactant. The biosurfactant of *B. velezensis* KLP2016 showed retention time (RT) 1.130 min (Fig. 4c), while the authentic surfactin and Iturin A showed a RT 1.27 min and 6.066 min respectively (Fig. 4a, b). Thus, the purified biosurfactant appeared to be a ‘surfactin-like’ biosurfactant molecule. The MS/ MS values of the peak (1058.60, 1044.62 and 1030.63 m/z) of the purified biosurfactant of *B. velezensis* KLP2016, were found similar to that present in commercial grade surfactin (Fig. 5a, b). On the basis of previously published literature [28], the purified biosurfactant produced by *B. velezensis* KLP2016 was reported as surfactin with *Mr* (~ 1.0 Dalton) as revealed by ESI–MS spectral analysis.

### Biodegradation of Engine oil (K 15 W-40) using CO_2_ stoichiometry analysis in a biometric system

**Fig. 6:**
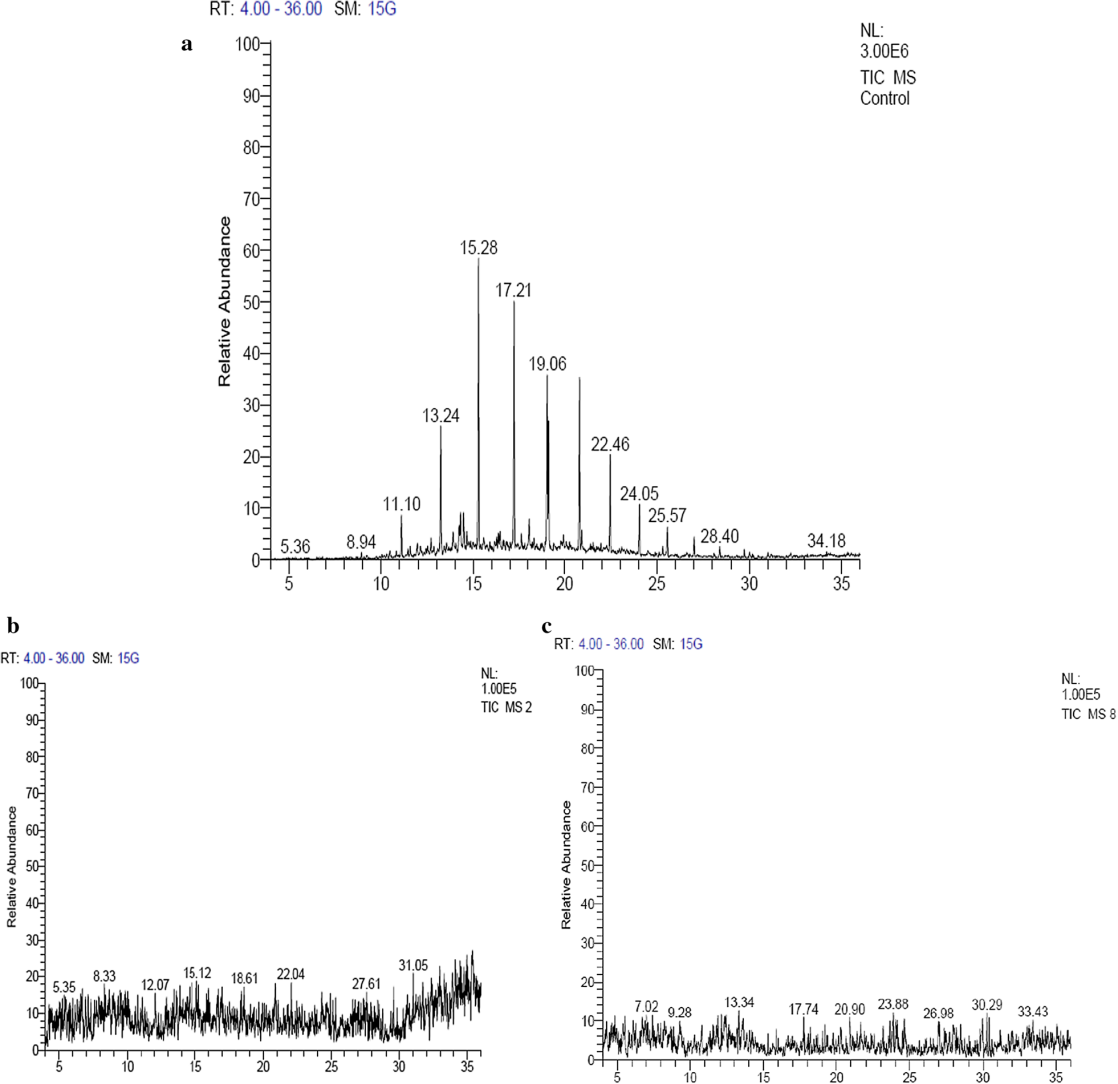
GC–MS analysis of Engine oil (supplemented in LB and MSM broth) without treatment and at 5 and 20 days of treatment with *B. velezensis* KLP2016. **a** GC–MS spectra of Engine oil (without treatment); (**b**) 5 days treatment of engine oil with *B. velezensis* KLP2016 in LB; and (**c**) 15 days treatment of engine oil with *B. velezensis* KLP2016 in LB medium

Bacterium-inoculated MSM and LB broth gave optical density 1.762 and 2.901, respectively, after 15 days of incubation (Table 1). Engine oil degradation was confirmed by the GC–MS analysis, which indicated disappearance of prominent peaks detected in engine oil (positive control) (Fig. 6a). Results showed that *B. velezensis* KLP2016 cells degrade engine oil efficiently when grown in LB broth after 15 days of incubation compared to MSM broth (Fig. 6c). The maximum carbon dioxide content trapped in the KOH solution after 15 days of incubation in LB and MSM broth showed values of 1980 × 10^−2^ ppm and 825 × 10^–2^ ppm, respectively (Table 1). Thus, LB broth was found as a better nutrient source for bacterial growth in context to engine oil degradation because a higher amount of CO_2_ was released then got trapped in KOH.

i. 2KOH + CO_2_ = K_2_CO_3_ + H_2_O
ii. K_2_CO_3_ + 2 HCl = H_2_O + CO_2_ + 2KCl

**Table 1:** Growth of *B. velezensis* KLP2016 in MSM and LB broth containing engine oil at 30 °C in a shake flask culture

Incubation time (days)	(MSM broth + engine oil + *B. velezensis*)		(LB broth + engine oil + *B. velezensis*)	
	OD_600_ nm	Fixed carbon dioxide (ppm)	OD_600_ nm	Fixed carbon dioxide (ppm)
0	0.665	–	0.742	–
5	1.312	550 × 10^–2^	2.085	880 × 10^–2^
10	1.421	770 × 10^–2^	2.402	1320 × 10^–2^
15	*1.762*	825 × 10^–2^	*2.901*	1980 × 10^–2^
20	1.667	814 × 10^–2^	1.720	1969 × 10^–2^

One molecule of K_2_CO_3_ contains one molecule or 44 g of CO_2_. To calculate the CO_2_ trapped by the KOH solution, K_2_CO_3_ was titrated with HCl. As per reaction, it was observed that 2 molecules of HCl are required to neutralize one molecule of K_2_CO_3._ CO_2_ trapped after the 5^th^, 10^th^, 15^th^ and 20^th^ days of incubation in LB broth was observed as 880 × 10^–2^, 1320 × 10^–2^, 1980 × 10^–2^ and 1969 × 10^–2^ ppm, respectively.

### Hydrocarbon analysis of engine oil (K 15 W-40) by GC–MS

The uninoculated LB-broth containing engine oil exhibited more peaks than *B. velezensis* KLP2016 inoculated or treated engine oil, as revealed after 5 and 15 days treatment. Engine oil was broken down into methylsulfonyl, borane, pyridine, piperazine, octanamide, ethylene, diethyl propyl and benzenenamine, as revealed on the basis of variation in the peaks generated by GC–MS spectra (Fig. 6).

## Discussion

Due to the hazardous effects of engine oil and associated hydrocarbons, it is urgent need to find methods of controlling and biodegrading them to safeguard the environment and human welfare. Biosurfactants have been successfully used in cleaning up polluted areas at low cost and high efficiency [29]. Biosurfactant-mediated remediation of hydrocarbons containing engine oil is an eco-friendly approach, which is able to transform toxic substances into nontoxic compounds, and this technique is an effective technology for the treatment of soil and water contamination [30]. In earlier reports, many methods for screening biosurfactants have been discussed, such as the haemolytic assay, BATH assay, oil spreading, drop collapse and surface tension measurement [31]. In earlier reports, these methods have been noted as screening methods, excluding surface tension measurement which is the key parameter for detecting surfactant activity [32]. Oil spreading is a widely used and effective biosurfactant screening method to detect the potential biosurfactant-producing microbes in the mixtures [33]. This method is a rapid detection method, which can be applied when the activity/quantity of biosurfactant is low in the respective fermentation medium [34].

It was reported earlier that biosurfactants produced from *Lactobacillus* sp. with molasses as substrate exhibited high surface tension reduction from 72 mN/m to values ranged from 47.50 ± 1.78 to 41.90 ± 0.79 mN/m with high emulsification activity ranged from 49.89 ± 5.28 to 81.00 ± 1.14% [35]. The surface tension of the biosurfactant containing cell-free broth of *Candida lipolytica* UCP0988 was reduced from 55 to 25 mN/m [36]. The ability to reduce surface tension was quantitatively determined by tensiometry, with 57 isolates which were found to lower culture supernatant surface tensions to 24.5–49.1 mN m^−1^ [37]. In our study, cell-free broth of the bacterium *B. velezensis* KLP2016 showed excellent biosurfactant properties, as was evident on the basis of emulsification activity, surface tension measurement and critical micelle concentration. All these methods strongly detected the biosurfactant nature of *B. Velezensis* KLP2016, as it reduced surface tension upto 40 mN.m^−1^ in an in vitro assay at 35 ºC after using cell-free broth of *B. velezensis* grown in LB broth.The critical micelle concentrations (cmc) of cell-free broth of *B. velezensis* grown in LB at 35 ℃ and 25 ℃ were 17.2 µg/mL and 17.4 µg/mL, respectively while in MSM broth at 35 ℃ and 25 ℃ were 17.6 µg/mL and 18.1 µg/mL respectively. The present results of cmc were found to be lower than the earlier reports [38, 39], who observed cmc (40 mg/L) for both *Bacillus subtilis* MG495086 and *Bacillus nealsonii* S2MT. E_24_% of the cell-free broth of *B. velezensis* was observed as 65.7%, 59.0%, 56.1%, 61.0%, 52.3%, 65.2% and 56.2% against benzene, pentane, cyclohexane, xylene, *n*-hexane, toluene and engine oil, respectively. The results of E24% were found to be higher than the earlier report [40].

Bacterial biosurfactants are generally peptides containing small lipidic moiety and gel permeation, hydrophobic interaction and ion exchange methods are generally employed for the purification from cell-free fermentation broth of *B. velezensis* KLP2016. In previous studies, ion exchange chromatography has been reported for the purification of biosurfactants [33]. Another lipopeptide-like biosurfactant was purified by using DEAE anion exchanger chromatography, followed by an HPLC [41] or a HiTrap Q system [24]. In the earlier reports, molecular sieve chromatography was also used to resolve the low molecular mass biosurfactant by using Sephadex as the matrices [24]. Ion exchange chromatography is also very effective in eliminating coloured contaminating molecules from the biosurfactant fraction, and this technique resolved the antibiotic biosurfactant peak from other chromatographic peaks [26].

UV–Visible spectrophotometry (210 nm) and thin layer chromatography (R_f_ 0.90) confirmed the purity of a biosurfactant molecule. A prominent single peak appeared during HPLC indicated the purity of the surfactin type biosurfactant produced by *B. velezensis* KLP2016. Furthermore; the ESI–MS data confirm the *Mr* ~ 1.0 kDa of the purified surfactin-type biosurfactant. The purified surfactin biosurfactant molecule exhibited higher engine oil degradation ability as compared to previous reports [19]. It was reported earlier that adaptation of microbial communities to hydrocarbons increases their hydrocarbon degradation rates [41]. According to previous studies, biosurfactants in oil-polluted soil can emulsify the oily hydrocarbon compounds by enhancing solubility and decreasing surface tension [24]. Crude biosurfactant of *B. nealsonii* S2MT remediate 43.6 ± 0.08 and 46.7 ± 0.01% heavy oil-contaminated soil at 10 and 40 mg/L dosage of crude biosurfactant, respectively [42]. The highest value of CO_2_ was recorded to be 1980 × 10^–2^ ppm, which was trapped in the KOH solution after 15 days of incubation of *B. velezensis* grown in LB broth containing 1% engine oil. Our results on engine oil degradation study was found to be ~ 1000 times higher than the previously reported value of 656 µmol [19], exhibiting the efficiency of the biosurfactants produced by *B. velezensis* strain.

The LB broth appeared to be the best nutrient source to sustain bacterial growth as well as providing an efficient adjustment of engine oil for the degradation, which was qualitatively and quantitatively analysed by GC–MS on the 5th and 15th days. Our results showed that there was ~ 75% more engine oil degradation by *B. velezensis* KLP2016 cells in LB medium than when allowed to grow in MSM medium. After the purification and characterization the bioactive biosurfactant is considered to be as a surfactin-like molecule.

## Conclusion

In the present investigation, surfactin biosurfactant, a member of lipopeptide family, was isolated from *Bacillus velezensis* KLP2016 (Accession number KY214239) and characterization by ESI–MS and HPLC. The biosurfactants exhibited surface tension reducing, emulsifying activity with engine oil degradation capability. Such biosurfactant-based approach towards engine oil degradation is highly promising and may play pivotal role in reduction of soil and water pollution in near future.
